# Proposed National Convention

**Published:** 1845-12

**Authors:** 


### THE PROPOSED NATIONAL CONVENTION.

We noticed, in the number of this Journal for Oct., the recommendation of the Medical Society of the State of New York, for holding a National Medical Convention in the city of New York, on the 1st Tuesday of May, 1846, for the purpose of "elevating the standard of medical education in the United States."

From the favorable notices in various medical journals, such as those of New York, Boston, Buffalo, Louisville, St. Louis, and New Orleans, we infer that the proposition is extensively approved by the profession. Several of the journals named have suggested topics for the consideration of such a convention, such as the establishment of a uniformity in the fees; requirements, &c., of medical schools; medical legislation; the formation of a national medical association, &c. While we are disposed to approve the object, and aid, if possible, its advancement, we share the doubts of the editor of the *New York Journal of Medicine*, in regard to its practicability and success. "We have," he says, "always advocated a higher standard of medical attainment for graduating in medicine, and a sufficient preparatory education to place physicians on a par with other learned professions; but we have seen so much of the levelling system; so much pandering to popularity; such audacious promises on the part of medical schools to gull pupils; such pretensions to cheapness in board; such mock examinations for degrees; such drumming up of students; and such underbidding in the price of tickets; in short, such artifices, and tricks, and measures, for the sake of putting a few dollars in the pocket; that we have almost lost our early faith in the practicability of medical reform; at least to that extent to which it ought to be carried in order to accomplish the desired end." Still, we *hope* for better results, and would, towards their attainment, suggest some views, which we have long entertained, and we have not seen presented by others for the consideration of the convention.

We have long thought that the establishment of the system of *concous*, or public trials before some competent tribunal, where the qualifications of candidates should be tested, and their relative merits decided upon, (the course pursued in France,) would be a remedy for most of the evils complained of in medical schools and the profession.

1st. It would prevent the multiplication of institutions. Persons whose situations do not permit of their competing successfully before the Boards of Trustees, who fancy their claims overlooked, and have no tribunal of appeal, establish rival schools; and thus originates one of the evils of which complaint is made.

2d. It would give the appointment of professors into the hands of medical men, for of such, chosen by medical bodies, should the Boards be composed. This power is now exercised by persons of different pursuits, who are not competent judges of medical acquirement.

3d. It would render such institutions as adopted it, *national.* Their teachers being chosen by medical bodies in different parts of the country; and their claims being open to all, would become the objects of ambition to all aspiring and worthy teachers.

4th. Being in the power of the profession, it is practicable. Legislation is not required to effect it. Nearly all schemes of improvement resting upon this, are, on that account alone, impossible.

In making this proposition, we are not influenced by any want of respect for teachers in medical schools as at present constituted. We know how many men of eminence, skill, and worth are among them, and do but give expression to an opinion long since formed and expressed, but which has attracted too little attention.

We would also recommend the adoption of a uniform national code of medical ethics, which might be of great service where medical societies are not in existence.

Among the plans suggested by others, there are two which have seemed to us particularly useful, that of Dr. Drake for the establishment of a national association, and that of Dr. Linton for a central board for granting diplomas. This latter, however, we would suggest should not supersede the schools, but confer a higher and an additional degree. D. B.

